# Role of Interleukin 6 in Acute Pancreatitis: A Possible Marker for Disease Prognosis

**DOI:** 10.3390/ijms25158283

**Published:** 2024-07-29

**Authors:** Alexandra Mititelu, Alina Grama, Marius-Cosmin Colceriu, Gabriel Benţa, Mihaela-Simona Popoviciu, Tudor Lucian Pop

**Affiliations:** 1 2nd Pediatric Discipline, Department of Mother and Child, Iuliu Hatieganu University of Medicine and Pharmacy, 400012 Cluj-Napoca, Romania; perta.alexandra1@gmail.com (A.M.); mariuscol92@gmail.com (M.-C.C.); gabi.benta@gmail.com (G.B.); tudor.pop@umfcluj.ro (T.L.P.); 2 2nd Pediatric Clinic, Emergency Clinical Hospital for Children, 400177 Cluj-Napoca, Romania; 3 Faculty of Medicine and Pharmacy, University of Oradea, 410073 Oradea, Romania; mihaelapopoviciu1965@gmail.com

**Keywords:** acute pancreatitis, IL-6, severity, prognosis, biomarker

## Abstract

Acute pancreatitis (AP) is a significant cause of morbidity, even in children, and is frequently associated with systemic manifestations. There are many cytokines involved in the inflammatory response characteristic of this disease. Interleukin 6 (IL-6) is one of the most important cytokines involved in AP, beginning from cellular injury and continuing to the systemic inflammatory response and distant organ involvement. IL-6 is a multifunctional cytokine that regulates acute-phase response and inflammation. It is produced by various cells and exerts its biological role on many cells through its high-affinity complex receptor. IL-6 has been investigated as a predicting maker for severe forms of AP. Many studies have validated the use of IL-6 serum levels in the first 48 h as a reliable marker for severe evolution and multisystemic involvement. Still, it has not been used in daily practice until now. This review discusses the main binding mechanisms by which IL-6 triggers cellular response and the AP pathogenetic mechanisms in which IL-6 is involved. We then emphasize the promising role of IL-6 as a prognostic marker, which could be added as a routine marker at admission in children with AP.

## 1. Introduction

Acute pancreatitis (AP) is a rare cause of abdominal pain with the possibility of severe evolution. In recent years, AP incidence has increased in adults and children [1,2].

The AP episodes are divided based on severity according to the revised Atlanta classification, which recognizes three forms of AP: mild (without local or systemic complications), moderate (with local complications like pancreatic/peripancreatic collections and/or organ dysfunction with resolution in less than 48 h), and severe (with organ dysfunction for more than 48 h or developing multiple organ dysfunction syndrome, MODS) [3].

Acute injury to the pancreas occurs after the exposure of acinar cells to various aggressions (gallstones, infections, trauma, toxins, or metabolites [4]. In daily practice, the existence of a pancreatic injury and its severity are monitored by performing specific tests (amylase and lipase). The increase in the serum level of these parameters confirms pancreatic suffering. It gives us an overview of the severity of the injury but does not provide a precise picture of the evolutive potential of the disease. For this reason, new markers that could predict the unfavorable evolution of AP early-on have been studied. Some scoring systems have been developed but are hard to apply and do not have a good predictive value [5]. Interleukin 6 (IL-6) is a biological marker that has been intensively analyzed to determine the severity of pancreatic damage, with promising results.

This review describes the involvement of IL-6 in the pathogenesis of AP and summarizes the data regarding using IL-6 as a prognostic marker for severe forms.

## 2. IL-6 and IL-6 Receptor

IL-6 is a multifunctional cytokine involved in many pathophysiological processes, such as infections; inflammation; and neuroendocrine, vascular, and malignant diseases [6,7,8]. It represents the primary molecule of the IL-6 family of cytokines, alongside nine other cytokines (IL-11, IL-27, IL-35, IL-39, oncostatin M, leukemia inhibitory factor, cardiotrophin 1, cardiotrophin-like cytokine factor 1, and ciliary neurotrophic factor) [7]. The serum levels of IL-6 in a physiological state are very low, but these levels increase extremely rapidly in the first phases of infections and inflammation [6].

Since the demonstration of the important role of the liver in acute-phase protein synthesis by Miller et al., the existence of some hormone-like molecules that can induce liver response has been proposed [9,10]. Initially, a monocyte-derived polypeptide involved in acute-phase protein synthesis was described and named hepatocyte-stimulating factor [9,10]. After the discrimination of IL-1 and TNFα from hepatocyte-stimulating factor, B-cell stimulatory factor 2 was described as a 26 kDa molecule and later named interleukin 6 [9,11]. The first strong evidence for the significant role of IL-6 in acute-phase reaction came from studies on cellular cultures. In human hepatocyte cultures, IL-6 determined the synthesis of C-reactive protein and serum amyloid in a dose- and time-dependent mode [9,12].

IL-6 represents a key mediator in the regulation and coordination of the immune response, with most immune cells being able to produce IL-6. In the initial stage of inflammation, IL-6 is synthesized locally. Subsequently, it quickly migrates to the liver, favoring the synthesis of acute-phase proteins (C-reactive protein, serum amyloid A, fibrinogen, haptoglobin, and α1-antichymotrypsin) [9].

Besides the effect on hepatocytes, IL-6 affects other cells, like B cells. IL-6 acts directly on B cells previously activated by IL-4 and IL-5 and leads to immunoglobulin A, M, and G secretion. IL-6 cannot act directly on normal resting B cells because they do not express IL-6 receptors [9,13,14].

There are two distinct pathways for the IL-6 action in the different cells (classical and trans-signaling). The first one is represented by the IL-6-specific cell receptors (IL-6R). This receptor has two subunits: an 80 kDa ligand-binding subunit (gp80) and a 130 kDa signal-transducing protein (gp130). The extracellular portion of the 80 kDa IL-6 receptor does not directly contribute to IL-6 binding. Still, it increases the affinity of the ligand for the gp130 subunit, which binds IL-6 and mediates the transduction of the signal. This mechanism is called the classical signaling pathway. It depends on both receptor subunits on the cell’s surface, which are expressed only on hepatocytes and certain subpopulations of leukocytes [6,15,16]. In the second pathway, IL-6 binds to a soluble form of the IL-6R (sIL-6R) and forms a complex (IL-6/sIL-6R) that increases the bioavailability of circulating IL-6, allowing its action on cells that lack IL-6R but express gp130. Thus, any cell that expresses gp130 can gain responsiveness to IL-6 through the trans-signaling pathway. Given that gp130 is largely expressed in immune and nonimmune cells, it explains the involvement of IL-6 in many pathologies. Both mechanisms activate the Janus kinase/STAT (JAK/STAT) signal transduction pathway [6,16]. The JAK/STAT pathways are important in immune response, contributing to the body’s defense against infections, maintaining immune tolerance, and strengthening barrier function [17]. JAK is a non-receptor tyrosine-protein kinase activated by different cytokines, which plays an important role in regulatory signal transmission. The JAK family has four main members (JAK1, JAK2, JAK3, and TYK2) expressed in almost all tissues. The STAT family is composed of seven members (STAT1, STAT2, STAT3, STAT4, STAT5a, STAT5b, and STAT6) [17].

**Figure 1:**
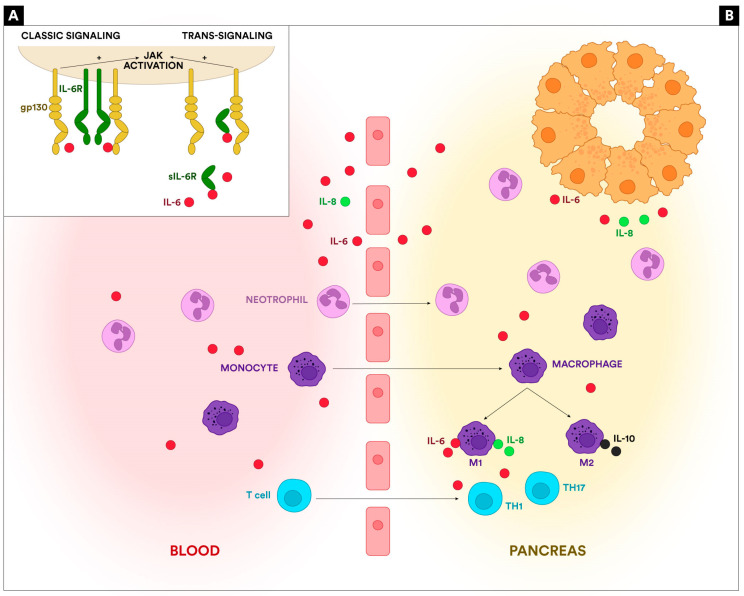
(**A**): The IL-6 signaling. The classic pathway can be activated on cells that express both IL-6R and gp130. The trans-signaling pathway occurs through a soluble form of IL-6R that binds IL-6 and forms a complex that can act on cells that lack the IL-6R but express gp130. Both pathways activate the JAK/STAT3 cascade. (**B**): The involvement of IL-6 in AP pathogenesis. IL-6 is released by the necrotic acinar cells, triggers the recruitment of inflammatory cells in the pancreas (neutrophils, monocytes, T lymphocytes), and activates the resident macrophages. IL-6 also determines the activation of Th1 and Th17 cells.

JAK/STAT is a common signaling pathway that transduces signals from the extracellular to the intracellular (nucleus). More than 50 cytokines, growth factors, and hormones are implicated in the JAK/STAT pathway, interfering with hematopoiesis, immune response, tissue repair, inflammation, apoptosis, and adipogenesis [17]. The two signaling pathways for IL-6 are represented in Figure 1A. 

## 3. IL-6 in the Pathogenesis of Acute Pancreatitis

The initiating mechanism in AP is the damage to the acinar cells. Necrotic acinar cells release damage-associated molecular patterns (DAMPs) and proinflammatory cytokines represented by IL-6, tumor necrosis factor (TNF-alpha), and interleukin-8 (IL-8), as well as anti-inflammatory cytokines, including interleukin-10 (IL-10). IL-6 is a multifunctional cytokine with pro- and anti-inflammatory properties and is one of the first cytokines involved in the inflammation associated with AP [15,18]. The cytokines released initiate the recruitment of phagocytes (neutrophils and macrophages) in the pancreatic tissue. IL-6 is closely involved in neutrophil migration by trans-signaling on endothelial, smooth muscle, and epithelial cells and by upregulating the expression of adhesion molecules like ICAM-1 and VCAM-1 [6,19]. After the neutrophil influx, IL-6 is involved in their burst, thus participating in the proinflammatory activity mediated by elastase, reactive oxygen species (ROS), and myeloperoxidase (MPO). More importantly, IL-6 is involved in neutrophil apoptosis, participating in the prevention of excessive inflammation [6,20]. IL-6 is leading to the shift from neutrophil recruitment in the pancreas to mononuclear cell recruitment and macrophage differentiation [6]. Macrophage differentiation is one of the most important mechanisms determining AP’s severity. The imbalance between M1 (proinflammatory) and M2 (anti-inflammatory) macrophage subtypes determines increased severity through extreme inflammation [21]. IL-6 plays a bivalent role: on the one hand, it is one of the cytokines secreted by M1-activated macrophages that induce a Th1-type response and inflammatory augmentation, secreting reactive oxygen species, chemokines, and cytokines; on the other hand, it can promote alternative activation of macrophages to the wound-healing phenotype [6,22]. The role of IL-6 in macrophage stimulation is not limited to the pancreatic resident macrophages. IL-6 and other proinflammatory cytokines migrate to the bloodstream and, through the portal vein, reach the liver and, afterward, the lung, activating Kupffer cells and alveolar macrophages, thus increasing inflammation and participating in the systemic progression of AP [22].

Furthermore, IL-6 plays an essential role in the acquired immune mechanisms involved in AP. IL-6 promotes the differentiation of naïve CD4 T-cells, thus being involved in the imbalance of the Th1/Th2 ratio, one of the most important mechanisms involved in AP severity [23,24]. IL-6 is also an important promotor of Th17 cell proliferation and IL-17 secretion, increasing inflammatory response, primarily through activating STAT3 in naïve CD4 cells [18]. The activation of the JAK/STAT signaling pathway via the signal transducing gp130 leads to the expression of various critical mediators of inflammation. It has been demonstrated that the JAK/STAT pathway activation promotes the progression of acute or chronic pancreatitis and can be the trigger for pancreatic tumors [25]. Another essential function of IL-6 is the stimulation of mature B cells, thus increasing levels of B-cell-activating factor (BAFF), an important marker of inflammation and severity in AP [6,26]. IL-6 is not only an inflammatory cytokine but also has anti-inflammatory action on T cells, stimulating the production of IL-10 [6]. The involvement of IL-6 in AP pathogenesis is shown in Figure 1B.

## 4. IL-6 as a Prognosis Marker for Acute Pancreatitis

Predicting the severity of AP episodes is one of the most critical concerns due to the high mortality and complications associated with severe, necrotic forms. There are several scoring systems developed for adults that can be useful but do not have sufficient accuracy. Another inconvenience of these scores is that they cannot be used in the pediatric population. Thus, finding a good marker with a high predictive value that is easy to use in children is desirable [27]. IL-6 is one of the most studied cytokines for this purpose. Since IL-6 is released beginning with the phase of cellular injury, it is one of the first inflammatory markers to be detected in the blood.

A study conducted by Dambrauskas et al. on 108 patients with AP demonstrated the utility of IL-6 as a prognostic marker compared with other cytokines. The evaluation of these markers was made at admission and was compared to the evolution based on imaging findings on CT on days four and seven. This study analyzed the prediction value of five cytokines (IL-6, IL-8, IL-10, MIF—macrophage migration inhibitory factor, and LTB4- leukotriene B4) for the local and systemic complications and fatal outcomes. IL-6 and MIF had the best predicting value in discriminating mild from severe forms, as well as the development of systemic complications (systemic inflammatory response syndrome—SIRS and multiple organ failure—MOF) and fatal outcomes. IL-6 was superior to MIF in differentiating edematous from necrotic forms [28].

Another study conducted by Ceranic et al. showed the superiority of IL-6 compared to other cytokine and severity scores. The study included 96 patients with AP. Severity scores (BISAP and Ranson) were calculated, and IL-6, IL-8, and IL-10 were measured at admission and after 48 h. IL-6 had a higher predictive value for severity at admission but also in the 48-h follow-up compared to IL-8 and IL-10. Furthermore, compared to the Ranson score, IL-6 measured at 48 h after admission exhibited similar predictive values for severe evolution [29].

Sternby et al. investigated the evolution of seven markers (IFN-γ, IL-1β, IL-6, IL-8, IL-10, IL-12, and TNF-α) over the first 48 h from the onset of the disease. This study concentrated on comparing the mean value of these parameters at the beginning and after the first 24 h of onset to identify the best timing for their predictive value. There were 115 patients included, classified as mild (71), moderate (33), and severe (11) according to the modified Atlanta criteria. All parameters except TNF-α had significant differences between the mild and severe groups. Still, only for IL-6, the delta values (the difference between the first and second mean values) varied significantly between all three severity groups. The main finding of this study was that the mean value of each severity group differed considerably after 24 h for IL-1β, IL-6, IL-8, and IL-10, with the most evident rise for IL-6. This may be useful for the identification of the best timing for the evaluation of these prognostic markers [30].

Kolber et al. also investigated the usefulness of IL-6 as a predicting marker for severity at admission and after 24 h. They enrolled 95 patients with AP, classified according to modified Atlanta criteria: twenty-nine mild, fifty-eight moderate, and eight severe. This study did not find significant differences between IL-6 levels upon admission and after 24 h of hospitalization. Patients with SAP had significantly higher levels of IL-6 on both days compared to the moderate and mild groups. Moreover, higher levels of IL-6 were observed in patients who developed necrosis. The level measured on the second day correlated with hospital length. There was no significant difference between the prediction accuracy of IL-6 compared with multi-variable scores (BISAP, Ranson’s, or APACHE II). This is a very important finding, given that these scores are sometimes hard to determine or cannot be applied, especially if we refer to the pediatric population [31].

The study conducted by Jain et al. demonstrated the utility of IL-6 in association with SIRS for increased precision in detecting severe cases. The study was conducted on 115 patients with mild, moderate, and severe AP. SIRS was diagnosed in the first 72 h of the onset of the disease, and additionally, IL-6, TNF-α, IL-10, MCP-1, GM-CSF, and IL-1β were measured. The study revealed that the combination of SIRS at admission and IL-6 >160 pg/mL had a significantly higher PPV than SIRS alone (85% vs. 56%) and a specificity of 95%. From the other cytokines, only MPC-1 showed good results in differentiating mild from severe forms, but none of them were able to increase the sensitivity in association with SIRS. The association of IL-6 measured at admission and SIRS can be an easy-to-use tool for the early identification of severe forms, especially in children, where the other scores of severity cannot be applied [32].

Tian et al. also investigated the usefulness of the combined detection of four serum markers. They included 153 patients with AP, 81 mild and 72 severe forms. CRP, PCT, IL-6, and LDH levels were measured at admission. Each of them showed significantly different levels between mild and severe forms. However, combining these four parameters measured at admission, the sensitivity and specificity for the early detection of severe forms were significantly increased. This combination could be a simple and cost-efficient test for early severity stratification [33].

Many other studies have evaluated the value of IL-6 as a marker for prognosis in AP, and the results are promising (Table 1). IL-6 serum levels are significantly elevated in patients with AP compared with controls, and there are differences between mild and severe forms [34,35,36,37,38,39,40,41,42,43,44,45,46,47]. IL-6 levels are higher in patients with distant organ failure and MOSF (multiple organ system failure) [44,45,46,47,48]. Some studies have shown a better predictive value for severe forms than other serum markers (CRP, IL-8, IL-1, IL-10, and TNF) [49,50,51,52,53,54,55,56,57,58,59]. Other studies have shown an increased sensibility for the association of IL-6 with other markers like CRP or PCT [60,61,62,63,64].

Although there are other inflammatory markers, like CRP, that are more available and already used for severity prognosis in AP, many studies show a superior predictive value of IL-6, especially in the very early stages [43,53]. It was observed that CRP peaks at 72 h of admission, having a delay of 24-48 h compared to IL-6, making IL-6 a more precocious marker [34,36]. CRP had a relatively high value in sensitivity and accuracy after the second day of admission in predicting severe forms, necrosis, and mortality [44,54]. The study conducted by De Beaux et al. showed there was no significant difference in the median serum levels of CRP on the first day of admission among patients who developed organ failure or a local pancreatic complication and those who had mild disease. By the second day of admission, the median serum level of CRP was greater in patients who developed organ failure than in those with a local pancreatic complication (*p* = 0.02) or with mild disease alone (*p* = 0.003) [48]. All these data, alongside other studies showing the usefulness of the combined assessment of IL-6 and CRP, concluded that IL-6 is not a replacement for CRP but rather an earlier or a complementary marker.

## 5. Limitations and Future Directions

As shown above, numerous data support the usefulness of IL-6 as an early predictor for severity in AP. Still, it has not been introduced in clinical practice because of some limitations. Firstly, IL-6 is involved in the very first stages of AP, and it is not stored in cells, meaning that it could have a rapid increase but also a decrease in serum levels. Thus, the measurement of serum levels has to be done close to the onset of the symptoms. In clinical practice, this may prove difficult, as it is sometimes hard to determine the exact timeline. Although all studies discussed above demonstrated the utility of IL-6 as a severity predictor, the timing for measurement is not very well-established. The majority of data indicate the first 48 h after admission as the best timeframe.

Secondly, there are no clear cut-off values. Some of the studies did not evaluate this parameter, or in the studies that are present, there are extremely large variations.

Moreover, the cost and availability of cytokine measurement is another limitation for routine use in daily practice. Although in the past this was the primary limitation, currently more and more methods are widely available with moderate costs. Furthermore, a closer analysis of the other severity scores shows us that they are time-consuming and need other laboratory parameters and imaging evaluation. At the same time, IL-6 measurement is rapid and can be used in children as well as adults.

Serum IL-6 measured in the first 48 h of admission could be used in clinical practice as a single parameter or as part of a new score associated with other serum markers. A point-of-care test for IL-6 has been used in different diseases and could be used in AP for decision-making and treatment strategy. More data is needed to establish unitary cut-off values for severity stages and systemic complications.

## 6. Conclusions

Severity stratification and prediction of severe, necrotic forms of AP is essential in managing the disease due to its rapid and unpredictable evolution. A reliable serum marker or a combination of markers could be the best approach for more standardized care. IL-6 is the most promising marker, with the best results, and should enter the panel of markers analyzed in patients with AP, including children.

## Figures and Tables

**Table 1:** Summarization of the evidence regarding the importance of IL-6 as a predictor marker for AP.

Authors	Year	Description	Results
Inagaki et al. [34]	1997	12 patients: 7 with mild pancreatitis, 5 with severe pancreatitis IL-6 measured 5, 24, 72, and 120 h after admission	There is a significant difference in IL-6 levels between mild and severe pancreatitis at all moments measured. The peak of IL-6 in the severe group was observed at 5 h after admission, 58.9 ± 18.8 pg/mL.
Pooran et al. [35]	2003	36 patients with AP and age-matched controls IL-6 measured 24 h of admission	There is a significant difference in IL-6 levels between controls and mild pancreatitis, controls and severe pancreatitis, and mild and severe pancreatitis. The average serum concentrations of IL-6 were 13.70 ± 1.90 pg/mL, 40.37 ± 4.35 pg/mL, and 82.76 ± 6.98 pg/mL in the control, mild pancreatitis, and severe pancreatitis groups, respectively. The difference in the means between control and mild pancreatitis groups was 26.67 pg/mL, between control and severe pancreatitis was 69.09 pg/mL, and between mild pancreatitis and severe pancreatitis was 42.39 pg/mL.
Berney et al. [36]	1999	43 patients with AP: 24 mild AP, 19 severe AP IL-6, IL-8, IL-10, CRP, and PMNE measured at admission and after 1, 2, and 5 days	High levels of IL-6 were detected in both groups upon admission (day 0). Higher levels of IL-6 were detected in patients with severe AP during the first two days (days 1 and 2) compared with those with mild AP. By day 5, IL-6 returned to low concentrations in both groups. The mean values for the mild group were 861 ± 20 pg/mL, 82 ± 28 pg/mL, and 54 ± 13 pg/mL, corresponding to days 0, 1, and 2 of admission. The mean values for the severe group were 33.600 ± 17.600 pg/mL, 486 ± 157 pg/mL, and 579 ± 184 pg/mL on days 0, 1, and 2. A 24 to 48 h delay was observed between peaks of IL-6 and CRP.
Kostic et al. [37]	2015	52 patients with AP: 34 mild and 18 severe, necrotizing AP IL-6, IL-8, IL-10, and TNF-α measured on days 1 and 3 after admission	Significantly higher IL-6 concentrations were detected in patients with necrotizing AP compared to those with interstitial AP on both days measured.
Viedma et al. [38]	1992	24 patients with AP: 9 mild AP, 15 severe AP IL-6, CRP, and phospholipase A measured at admission and every day until day 7	Higher IL-6 levels were detected in patients with severe compared with mild AP in the first two days and also in those with necrotizing AP compared with non-necrotizing AP.
Park et al. [39]	2015	59 patients with AP: 36 mild AP and 23 moderate AP IL-6, IL-Iβ, IL-1ra, TNF-α, sTNFR-I, and sTNFR-II were measured at admission and after 24 h	Significantly higher levels of IL-6 were detected in moderate compared to mild AP in both days of measurement. In patients with mild AP and with moderate AP, the mean levels of IL-6 on day 2 were 91.1 ± 230 pg/mL and 163 ± 170 pg/mL, respectively. Higher IL-6 levels were also detected in obese patients compared with non-obese. The mean levels of IL-6 on day 2 in overweight and non-overweight patients were 126 ± 124 pg/mL and 115 ± 244 pg/mL, respectively.
Gunjaca et al. [40]	2012	20 patients with AP: 13 mild and 7 severe AP IL-6 measured on days 1, 2, 3, 6, and 9	There is a significant difference in IL-6 levels between mild and severe AP (23.4 pg/mL vs. 539.2 pg/mL, *p* < 0.0001)
Riché et al. [41]	2003	48 patients with necrotizing AP: 15 infected, 33 non-infected IL-6, CRP, PCT, and TNF-α were measured during the first 3 days	Higher IL-6 levels were seen in patients with infected necrosis compared with those without infection IL-6 < 400 pg/mL and PCT < 2 ng/mL combined identified best the patients who were not at risk for necrosis infection
Kumar et al. [42]	2022	62 patients with AP: 40 mild, 22 severe forms IL-6 and CRP were measured in the first 24 h of admission and also 48 h after admission	There is a significant difference in IL-6 and CRP mean levels between mild and severe AP. IL-6 at 48 h had the maximum AUC of 0.98. The cut-off values of IL-6 in days 1 and 2 for predicting SAP were 137 pg/mL and 77.3 pg/mL, respectively. IL-6 on day 1 and day 2 and CRP on day 2 were 100% sensitive, but IL-6 on day 1 and day 2 had a maximum specificity of 88.37% among them when compared with a specificity of 81.4% of CRP on day 2.
Leser et al. [43]	1991	50 patients with AP: 25 mild AP, 15 severe AP, 10 lethal AP IL-6 and CRP were measured daily for the first 7 days of AP	High IL-6 levels (with a cut-off value of 15 U/mL coresponding to 150 pg/mL) in the first two days of AP predicted (PPV 91%, NPV 82%) a severe or lethal course of the disease more accurately than elevated CRP. IL-6 is a better early parameter for the assessment of the severity of acute pancreatitis than CRP.
Khanna et al. [44]	2013	72 patients with AP IL-6, CRP, and procalcitonin measured on the first day of admission	IL-6 (values over 50 pg/mL) has the highest sensitivity for the prediction of SAP (93.1%), organ failure (95.7%), pancreatic necrosis (94.1%), and mortality (100%). The serum concentration of IL-6 on the first day and/or together with serum CRP concentration on the second day of admission is helpful in earlier prediction and assessment of the severity of acute pancreatitis, taking into consideration the disadvantages of multifactorial scoring systems.
Malmstrøm et al. [45]	2012	60 patients with severe AP IL-6, IL-8, IL-18, and TNF-α measured at admission and on days 1, 2, and 14	IL-6 values determined on days 1 and 2 can significantly predict organ failure (renal, respiratory, circulatory, and MOF) in the early stages. The median values of IL-6 in renal, respiratory, and circulatory failure were 840 pg/mL, 185 pg/mL, and 999 pg/mL, respectively, measured on the first day of admission.
Sathyanarayan et al. [46]	2007	30 patients with AP: 17 mild AP, 13 severe AP IL-6 measured on days 1, 3, 7, and 14 of admission	IL-6 was significantly higher on day 3 in severe AP than in mild AP (146.29 ± 57.53 pg/mL vs. 91.42 ± 71.65 pg/mL) and was significantly higher in patients who developed organ failure (161.59 ± 53.46 pg/mL vs. 88.16 ± 65.50 pg/mL). At a cut-off value of 122 pg/mL on day 3, IL-6 predicted organ failure and severe pancreatitis with a sensitivity and specificity of 81.8% and 77.7%, respectively.
Yao et al. [47]	2024	307 patients with AP: 260 with mild and moderate forms and 47 with SAP IL-6, IL-1β, IL-2, IL-4, IL-5, IL-10, IL-8, IL12p70, IFN-γ, IFN-α, and TNF-α were measured at admission	IL-6 had the best results in discriminating between mild and severe forms compared to the other cytokines studied. At a cut-off of 24.67 pg/mL, IL-6 can predict severe forms with a sensitivity of 87.23% and a specificity of 66.92%. Moreover, IL-6 predicted the occurrence of peripancreatic colections and necrosis.
De Beaux et al. [48]	1996	58 patients with AP: 30 mild AP, 28 severe AP IL-6, TNF-α and CRP were measured on the first and second days of admission.	Higher IL-6 levels were associated with severe AP compared to mild forms (*p* < 0.001) and organ failure development (*p* < 0.03).
Gregoric et al. [49]	2010	234 patients with SAP IL-6 was measured upon admission, and SIRS was calculated	SIRS at admission combined with IL-6 serum levels could be an accurate marker to predict severe AP (performing similarly to APACHE II and Ranson scores). IL-6 levels were significantly higher in the patients with a SIRS score of 3 or higher (72 +/− 67 pg/mL vs. 18 +/− 15 pg/mL).
Karpavicius et al. [50]	2016	102 patients with AP: 27 mild, 55 moderate, and 20 severe forms IL-6, adipokines, and CRP were measured at admission and on day 3 of hospitalization	IL-6 was the best predictor for severe forms. IL-6, with a cut-off value of 157 pg/mL, can significantly predict necrosis.
Pezzilli et al. [51]	1995	38 patients with AP: 13 mild AP, 15 severe AP IL-6, IL-8, CRP, and *β*_2_-microglobulin were measured on admission and daily for 5 days	IL-6 (cut-off 2.7 pg/mL) was the best marker for evaluating the severity of AP (sensitivity 100%, specificity 86%, diagnostic accuracy 91%).
Curley et al. [52]	1993	29 patients with AP: 16 mild AP, 13 severe AP IL-1, IL-6, IL-8, and IL-10 were measured at admission and after 48 h	Significant increases of IL-6 (69.5 pg/mL versus <10 pg/mL, *p* = 0.01) in severe compared with mild AP.
Pezzilli et al. [53]	1999	40 patients with AP: 25 mild AP, 15 severe AP Lipase, IL-6, and CRP were measured at admission	IL-6 (cut-off 3.7 μg/L) had a sensitivity of 100%, specificity of 83%, and AUC of 0.91 in discriminating severe from mild AP. C-reactive protein (cut-off values ranging from 6 to 7 mg/L) showed a lower prognostic efficiency than interleukin-6: sensitivity of 87% (13 of 15) and specificity of 46% (11 of 24).
Jiang et al. [54]	2004	33 patients with AP: 19 mild AP, 14 severe AP IL-6, CRP, and TNF-α were measured on days 1, 2, 3, and 7	Significantly higher IL-6 levels were detected in severe compared with mild AP (sensitivity 100%, specificity 89.7%, and accuracy 91% on day 1).
Rao et al. [55]	2017	40 patients with severe AP IL-6 measured in the first 24 h of admission	IL-6 (≥28.90 pg/mL) was the most accurate marker (compared with IL-8, IL-10, and CRP) for predicting the progression to severe pancreatitis.
Mayer et al. [56]	2000	51 patients with AP: 16 mild AP, 35 severe AP Il-6, IL-1β, IL-8, IL-10, IL-1RA, and sIL-2R were measured daily for 7 days after admission	IL-6 was significantly elevated in patients with distant organ failure and was the best prognostic parameter for pulmonary failure with median values between 989–2071 ng/mL (compared with the other markers investigated). IL-6 reached its highest serum concentration on day 3 after the onset of symptoms
Chen et al. [57]	1999	50 patients with AP: 23 mild AP and 18 severe AP TNF-α, IL-1β, IL-6, IL-8, and CRP were measured on days 1, 2, 3, 4, and 7	Interleukin-6 in day 1 of admission was the most useful prognostic biomarker, compared with the other markers investigated. The sensitivity, specificity and accuracy of predicting a severe attack were 89%, 87%, and 88%, respectively, using IL-6 >400 pg/mL on day 1.
Fisic et al. [58]	2013	150 patients with AP: 122 mild AP, 28 severe AP IL-6, IL-8, IL-10, and sTNFr were measured on the first day of admission	IL-6 is the best predictor for severe forms and systemic complications in patients with AP compared with other markers (IL-8, IL-10, and sTNFr). IL-6 greater than 37.9 ng/mL can be considered high-risk in terms of developing systemic complications with a sensitivity of 82% and a specificity of 65%.
Bidarkundi et al. [59]	2002	30 patients with AP: 15 mild AP, 15 severe AP, and 15 controls The percentages of PBMCs (peripheral blood mononuclear cells) that contained IL-6 and IL-12 were measured at admission and compared with APACHE III	Higher IL-6 levels were detected in patients with severe compared with mild AP. Values of 25% for IL-6-positive PBMCs and 30 for APACHE III score were the best cut-off values to differentiate mild from severe AP. With IL-6 values >25%, both sensitivity and specificity were 100%, whereas APACHE scores of >30 showed a sensitivity of 80% and specificity of 100%.
Cho et al. [60]	2023	103 patients with AP: 42 mild, 53 moderate, and 8 severe AP IL-6, CRP, and procalcitonin were measured at admission, 24 and 48 h after; CTSI was calculated at admission	IL-6 with a cut-off <50 pg/mL can predict mild forms with an accuracy of 83.3% at admission. The accuracy decreases slightly at 24 and 48 h of admission.
Chen et al. [61]	2020	90 patients with AP: 30 mild AP, 30 severe AP, 30 recurrent AP IL-6, TNF-α, IL-1, IL-8, IL-10, and miRNAs were measured at admission	Combined determination of serum IL-6 and hsa-miR-126-5p may be useful for the early prediction of AP classification (AUC, 0.991; sensitivity, 93.3%; specificity, 96.7%; cut-off value, 0.802; *p* < 0.001)
Nieminen et al. [62]	2014	163 patients with AP: 103 mild AP, 35 moderate AP, 25 severe AP 48 cytokines, including IL-6 and growth factors, were measured at admission	IL-6 (with a cut-off of 501.6 pg/mL), HGF, and their combination were prognostic biomarkers for severe AP (sensitivities of 56%, 60%, and 72%, respectively, and specificities of 90.6%, 92.8%, and 89.9%, respectively).
Sternby et al. [63]	2017	175 patients with AP: 124 mild AP, 41 moderate AP, 10 severe AP 20 biomarkers were measured, including IL-6, at 13–36 h after admission	IL-6 (cut-off 23.6 pg/mL) and CRP (cut-off 23.6 mg/L) combined demonstrate a clinically relevant capacity (AUC 0.803) to differentiate non-mild from mild AP in early stages.
Li et al. [64]	2022	67 patients with AP: 10 mild AP, 22 moderate AP, 35 severe AP IL-6 and CRP were measured on the first day after admission	The AUC of IL-6 (cut-off = 121.1 pg/mL) measured within 48 h of onset for the prediction of SAP was 0.69 (95% CI, 0.56–0.82), with a sensitivity of 67.65% and a specificity of 67.74%. IL-6 was more accurate than CRP for predicting mortality (AUC 0.70 vs. 0.75) and infected pancreatic necrosis (AUC 0.65 vs. 0.81) in patients with AP.
Farrell et al. [65]	2021	66 children: 36 mild AP, 10 severe AP, and 30 controls IL-6, MCP-1, and CRP were measured at admission	Levels of interleukin-6 (IL-6) and MCP-1 were statistically different between the mild acute pancreatitis and severe acute pancreatitis groups (*p* = 0.02 for both). IL-6 resulted in a significant ROC curve (AUROC 0.83 [95% CI, 0.67–1.00], *p* = 0.03) for predicting severe forms.

## Data Availability

No new data were created or analyzed in this study.
